# The Role of Meaning in Life Within the Relations of Religious Coping and Psychological Well-Being

**DOI:** 10.1007/s10943-014-9983-3

**Published:** 2014-12-19

**Authors:** Dariusz Krok

**Affiliations:** Institute of Family Sciences, Opole University, ul. Drzymały 1a, 35-342 Opole, Poland

**Keywords:** Meaning in life, Religious coping, Psychological well-being, Purpose and significance

## Abstract

The purpose of this study was to examine whether meaning in life understood in terms of presence, search, and personal meaning is a mediator in the relationships between religious coping and psychological well-being. Associations of religiousness and psychological well-being are complex and suggest the existence of meaning and purpose in their internal structures. Two studies were conducted. In Study 1, presence of meaning in life was a mediator between negative coping and psychological well-being in the scope of a total score and all its dimensions. Search for meaning in life did not mediate the above relations. In Study 2, personal meaning turned out to be a partial mediator between negative coping and psychological well-being. These findings suggest that meaning in life is a crucial element of religious coping and psychological well-being that is used by people as a part of their meaning system to cope with life’s difficulties and challenges.

## Introduction

### Conceptualizing Meaning in Life

One of the factors that affect human functioning is meaning in life. Many psychologists consider it to be a vital part of mental processes and behaviour (Frankl 1979; Steger 2012; Wong 1998). It enables individuals to interpret and organize their daily experience, achieve goals, and categorize important objects. Despite widespread agreement regarding the importance of meaning in life, defining it poses a challenge from both a theoretical and empirical point of view. Frankl (1965) in his theory of meaning proposed that each person has some unique purpose or overarching aim for their lives and tries to actualize as many values as possible in their community. Baumeister (1991) emphasizes that meaning in life depends on purpose, efficacy, value, and self-worth. Emmons (2003) defined meaning in terms of purpose and goals that are pursued by individuals. Other researchers describe meaning in life in terms of significance (Yalom 1980). People can experience meaning in life when their lives make sense or convey significant and comprehensible information.

Analysing various approaches to meaning in life Steger (2009) defines meaning in life as “the extent to which people comprehend, make sense of, or see significance in their lives, accompanied by the degree to which they perceive themselves to have purpose, mission, or overarching aim in life” (p. 682). This definition is broad enough to encompass a wide range of human experiences related to purpose and significance and also suggests that people can find meaning by engaging in creative endeavours and pursuing their goals. Steger (2012) points out that meaning is a superordinate term that encompasses two main factors: comprehension and purpose. The first factor reflects the human ability to make sense of and understand one’s life, including one’s self, the external world, and how one fits with and operates within the world. The second factor denotes overarching and long-term life aspirations that are self-concordant and stimulate related activity.

According to Steger et al. (2006), people’s approach to understanding meaning in life can be viewed in two principal dimensions: presence of meaning in life and search for meaning in life. The former concerns the degree to which individuals perceive their lives as significant and meaningful. The latter reflects the degree to which people are engaged in a search for meaning in life. Both dimensions have an essentially different character and express dissimilar attitudes to life. Presence of meaning in life allows people to experience their lives as comprehensible and significant and feel a sense of purpose or mission in their lives that transcends the mundane concerns of daily life (Steger 2009). Search for meaning in life relates to the dynamic and active efforts individuals expend when they try to comprehend the meaning, significance, and purpose of their lives (Steger et al. 2008). Some researchers pointed out that searching for meaning is a basic human motivation (Frankl 1965), whereas others stressed the importance of the presence dimension (Reker 2005; Reker and Wong 1988).

The presence of meaning in life approach was meticulously examined by Wong (1989) who defined meaning in life in the category of personal meaning as an individually constructed cognitive system, which endows life with personal significance. It can be conceived as a dynamic, cognitive map that directs people through their life course. According to Wong’s (1998) implicit theories research, the meaning system consists of five components: affective, motivational, cognitive, relational, and personal. The categories are used to assess both self and life events in regard to meaning (McDonald et al. 2012). Personal meaning measures implicit meaning (i.e. conceptions and beliefs regarding life held by people) which is viewed as “comprising an ideally meaningful life”. It assesses the level of meaning in seven domains: achievement (pursuit and attainment of significant life goals), relationship (general social adeptness), religion (having affirmative beliefs about relationships with the divine), self-transcendence (engagement in selfless pursuits), self-acceptance (a humble acceptance of one’s limitations), intimacy (having emotionally close relationships), and fair treatment (perceiving a degree of justice in life).

### Relations Between Religion and Meaning in Life

Many studies have been conducted which repeatedly show that religion can be a powerful source of meaning in life (Chamberlain and Zika 1992; Emmons 2005; Park 2005, 2013). Religion can be defined as “a search for meaning in ways related to the sacred” (Pargament 1997, p. 32). Despite the fact that the relationship between religion and meaning is intimate and complex, many people find in religious beliefs a sense of purpose, understanding, and psychological support (Park 2013). In their seminal book *“*The psychology of religion. An empirical approach”, Hood et al. (2009) clearly state: “The assumptions forming the fundamental framework for this book are that the search for meaning is of central importance to human functioning, and that religion is uniquely capable of helping in that search” (p. 12). Religious systems provide individuals with an integrated set of beliefs, goals, and meanings which can be used in explaining intricacies of the world and dealing with personal situations and problems.

Analysing religion as a provider of meaning, Hood et al. (2009) emphasize four criteria by which religion is uniquely capable of providing global meaning: comprehensiveness, accessibility, transcendence, and direct claims. First, religion is the most comprehensive of all meaning systems, because it includes an extensive range of other sources of meaning, such as social relationships, work, family, achievement, personal relationships, and important values. Religion is accessible to people in the sense of contributing to global meaning through promoting doctrinal teachings and creeds, religious education, and ethical norms of acceptable and unacceptable behaviours. The next criterion: transcendence reflects people’s tendency to search for an invisible reality and explore “ultimate concerns” (Emmons 2005) which requires some belief in an ultimate authority in which higher meaning is found. Finally, religion makes direct claims to provide a sense of significance as it facilitates people’s efforts to interpret their experiences in terms of existential intentions and meaning which is embedded within religion’s sacred character.

The ability of religion to provide meaning in life is particularly noticeable in the context of coping with stress and major life events. Religious coping can be defined in terms of “the degree to which religion is a part of the process of understanding and dealing with critical life events” (Pargament et al. 2005, p. 482). The results of studies conducted by Pargament (1997) who introduced the concept of religious coping reveal that it can be helpful or harmful, depending upon the particular type of religious coping strategy employed. In general, religious coping methods can be grouped into two wide overarching categories: positive and negative religious coping (Pargament et al. 1998). Positive religious coping styles relate to secure relationships with God and a sense of spiritual connectedness with others. They tend to be more beneficial for people who experience stressful events. Negative religious coping styles reveal insecure relationships with God and strains between individuals, and as research indicates, they are usually maladaptive (Ano and Vasconcelles 2005; Pargament et al. 2004). In that sense, religion is more than a defence mechanism as it can provide individuals with comprehensive and integrated frameworks of meaning that enable them to explain difficult life occurrences in satisfactory way.

Research carried out by Park (2005) on bereaved college students shows that religious meaning may affect coping processes by making meaning after the loss of a loved one. In the initial period of mourning, people who had strong religious beliefs tended to experience more stress, which was linked to the discrepancy between their global meaning system and appraisals of such events. However, after a period of a few months, these effects disappeared or even reversed, suggesting a positive association between religion and long-term adjustment. Drawing on religion as a meaning system, studies show that individuals can derive comprehensive frameworks of personal meaning from their religious beliefs which in turn influence their abilities to cope with adversity and life stress (Park 2007, 2013). Some aspects of religion, especially intrinsic religiousness, appear to be positively associated with a sense of meaning in life (Ardelt 2003; Chamberlain and Zika 1992; Steger and Frazier 2005).

### Meaning in Life as an Important Factor for Well-Being

There is substantial evidence demonstrating that meaning in life is associated with well-being and positive functioning. People who believe that their lives have meaning are happier (Debats et al. 1993) and report a higher level of life satisfaction which reflects the degree to which individuals positively evaluate their lives (Chamberlain and Zika 1988; Steger et al. 2008). Studies have also confirmed links between meaning in life and various measures of global happiness (Ryff and Keyes 1995), psychological adjustment (Thompson et al. 2003), and general well-being (Ho et al. 2010). The results provide strong evidence that validates positive relations between meaning in life and domains reflecting positive functioning.

Meaning of life is also associated with positive affect and emotions which are often regarded as an emotional dimension of subjective well-being (Diener 2009). People reporting high levels of meaning tend to experience stronger positive affect (King et al. 2006) and less negative affect (Chamberlain and Zika 1988), depression and anxiety (Debats et al. 1993). In his meta-analysis, Pinquart (2002) concludes that the correlation between meaning in life and positive affect is .47. King and Hicks (2013) note that those correlations range from .45 between positive affect and a composite of PIL and SOC questionnaires (in a sample of 586 undergraduates) to .80 for positive affect and SOC (in a sample of 266 community adults).

A very promising area of research on meaning in life and well-being lies within the model of psychological well-being (PWB) that is explicitly concerned with the development and self-realization of the individual (Ryff 1989; Ryff and Keyes 1995). PWB has been defined as “engagement with existential challenges of life” (Keyes et al. 2002, p. 1007) and is arguably best represented by Ryff’s (1989) PWB model. The PWB model is based on a eudaimonic approach that perceives life in terms of virtue defined as finding the middle ground between excess and deficiency and conditioned upon self-truth and self-responsibility (Ryff and Singer 2008). According to Ryff (1989), this model of well-being includes six constructs: (1) self-acceptance, (2) positive relations with others, (3) autonomy, (4) environmental mastery, (5) purpose in life (PIL), and (6) personal growth. Taken together, they offer a multidimensional, eudaimonic vision of human life. Research on PWB shows that it is associated with more optimal human functioning and health benefits (Ryff and Singer 2008) and a stronger sense of coherence (Krok 2010). There were also found positive associations between meaning in life and PWB, suggesting that a sense of purpose and significance is related to personal development and self-realization (Krok 2011).

### Direct and Indirect Relations Between Religion and Well-Being

A growing body of research indicates that religion is linked to well-being. Studies conducted in many countries confirmed positive associations between religiousness and general well-being (Delbridge et al. 1994; Green and Elliott 2010; Silberman 2005). The connections were particularly observable among older adults (McFadden 1995), which supports the assumption that this category of people can rely more on religion as a source of comfort and significance. Examining the associations between religiousness and individual and communal well-being, Myers (2008) points out that actively religious individuals report distinctly greater happiness and life satisfaction in comparison with their non-religious counterparts. However, Diener and Clifton (2002) noticed that the results of large-scale surveys pointing to relations between various aspects of religiousness and well-being are quite modest. According to their findings, the correlation between religiousness and life satisfaction is rather small (*r* = .08) and between religiousness and happiness equals *r* = .06.

In order to explain this consistent positive correlation between religiousness and well-being, some researchers proposed that the relation can be mediated by such factors as meaning in life (Chamberlain and Zika 1992), social support and optimism (Salsman et al. 2005), and prosocial behaviour (Kim 2003). Others suggested that religiousness incorporating a sense of purpose and significance may lead to the assignment of more positive meanings to ordinary daily events, which may, in turn, generate positive emotions related to religious experiences (Park 2013).

Empirical evidence confirms this assumption. Chamberlain and Zika (1992) found that the association between religion and well-being is mediated by an enhanced sense of meaning in life. Examining effects of religion and PIL on older adults, Ardelt (2003) revealed that intrinsic religious orientation and religious involvement have an indirect effect, mediated by PIL, on subjective well-being. Intrinsically religious older people who have found a sense of meaning and PIL were more likely to participate frequently in spiritual activities with others and to belong to a religious group than were extrinsically religious people.

Results obtained by Steger and Frazier (2005) demonstrated that meaning in life mediated the relation between religiousness and well-being in a university student sample when well-being was assessed by life satisfaction and self-esteem. In addition, meaning in life turned out to be an important mediator of the relation between daily religious activity and well-being, which can suggest that religious individuals might feel greater well-being because they derive meaning in life from their religious feelings and activities. Krok (2010) confirmed a mediational role of meaning in life in relations between religious meaning system and PWB. However, research has not been conducted that would investigate relations between religious coping and PWB which could reveal more precisely the nature and mechanisms relating religion to well-being.

The evidence from the above studies indicates that in the context of other potential mediators between religiousness and well-being, meaning in life appears very promising. Yet, examination of measures of meaning in life reveals that these scales often refer to diverse dimensions and are based on different premises. Some of them assume that daily life itself may be assessed with regard to meaning [e.g. the PIL, Crumbaugh and Maholick 1964 and the Personal Meaning Profile (PMP), Wong 1998], while others evaluate meaning in life in terms of both presence and search (Steger et al. 2006). Thus, meaning in life is not simply a one-dimensional, global judgment made about a life as a whole but a construct that encompasses more dimensions. It needs to be examined from different perspectives if we want to discover the complexities of meaningful life and find variables associated with daily meaning in life.

### Overview of Studies

In the present study, we decided to examine meaning in life as a factor that can explain the relation between religious coping and PWB. First, we wanted to investigate more deeply relationships between religious coping and PWB which expand our knowledge about the ways in which people rely on their religious beliefs and activities in building existential life satisfaction and forming a global judgment of a well-lived life. People use their religion in order to discover the deeper truths and mysteries in life and to understand what makes their life valuable. Second, because meaning in life was analysed in the perspectives of both presence and search (Study 1), and personal meaning (Study 2), it allows us to investigate its different dimensions and their mediational effects between religious coping and PWB. Meaning in life is often portrayed as emerging out of motivation. Therefore, the question of whether its dimensions play a role in mediating relations between the forms of coping based on religious experiences and well-being ingrained in existential challenges of life appears very important. Finally, we wanted to assess which dimensions of meaning in life are most associated with PWB. It is very likely that when individuals have a view of life as personally significant and purposeful, they ought to experience their life as valued and self-fulfilled.

## Study 1

In Study 1, we examined how positive and negative religious coping related to meaning in life and PWB. Then, we used multiple regression and mediational tests to assess the degree to which presence of and search for meaning in life mediated the relation between the two ways of religious coping and PWB. We used PWB as an outcome measure because it has received considerable research attention and occupies a prominent place in theories of eudaimonic well-being (Ong and Zautra 2009).

The study focused on the following hypotheses: (1) endorsement of religious coping styles will be differently correlated with individuals’ meaning in life. That is, positive coping will show positive associations with meaning in life, whereas negative coping will correlate negatively with meaning in life; (2) there will be associations between religious coping and PWB; (3) presence of meaning in life will be a stronger mediator between religious coping and PWB than search for meaning in life.

### Method

#### Participants

A total of 187 participants (97 women and 90 men) completed a questionnaire packet consisting of three methods. Ages ranged from 19 to 62, with a mean age of 30 years (SD = 9.45). There were no statistically significant differences in terms of age between women and men. The vast majority of participants were Christians (91.8 %), with only 8.2 % describing themselves as non-believers (who nevertheless occasionally used religious coping strategies). The study was anonymous.

#### Measures and Procedure

Three psychological tests were administered: Brief RCOPE, the Meaning in Life Questionnaire (MLQ) and the Psychological Well-Being Scale (PWB). All of them were Polish versions.

Brief RCOPE is a 14-item questionnaire that assesses the extent to which the person uses specific methods of religious coping (Pargament et al. 1998). It consists of two subscales: (1) positive religious coping that measures seeking spiritual support, seeking a spiritual connection, collaboration with God in problem solving, religious forgiveness, and benevolent religious appraisals of illness; and (2) negative religious coping that assesses punishing God appraisals, interpersonal religious discontent, demonic appraisals, spiritual discontent, and questioning God’s powers. People indicate how often they engage in each form of religious coping on a 4-point scale from 0 (not at all) to 3 (a lot). This instrument has demonstrated good construct validity and internal consistency in the Polish version (Jarosz 2011).

The MLQ was developed by Steger et al. (2006) to assess two dimensions of meaning in life: presence and search. It consists of 10 items rated on a 7-point scale from 1 (absolutely untrue) to 7 (absolutely true). The presence subscale measures the extent to which participants perceive their lives as meaningful (e.g. “I understand my life’s meaning” and “My life has no clear purpose”). The search subscale measures the extent to which respondents are actively seeking meaning or purpose in their lives (e.g. “I am searching for meaning in my life” and “I am looking for something that makes my life feel meaningful”). The MLQ Polish version adapted by Krok (2011) was used.

The PWB scale is a 42-item questionnaire that evaluates the level of individuals’ development and self-realization (Ryff and Keyes 1995). It comprises six scales: autonomy, environmental mastery, personal growth, positive relations with others, PIL, and self-acceptance. Each scale consists of seven items, with a mix of positive and negative items. On a scale from 1 to 6, respondents indicated whether they agreed or disagreed strongly, moderately, or slightly that an item described how they thought and felt. As in Sheldon and Lyubomirsky’s (2006) study, a total PWB score can be calculated by adding all the six dimensions. The Polish version was adapted by Krok (2010).

Those who agreed to participate in the study completed a questionnaire packet containing demographic items, measures of religious coping, meaning in life, and PWB. The participants either returned the completed questionnaires directly or sent them back by post.

### Results and Discussion

As an initial examination of the predicted relations, correlations were computed among religious coping, meaning in life, and PWB, and these are shown in Table 1.

**Table 1:** Correlations among religious coping, meaning in life, and psychological well-being

	1	2	3	4	5	6	7	8	9	10	11
1	–										
2	.18*	–									
3	.23**	−.23**	–								
4	.22**	−.06	.19**	–							
5	−.04	−.32***	.28***	−.04	–						
6	.04	−.32***	.31***	−.02	.55***	–					
7	.10	−.27***	.37***	.15*	.40***	.48***	–				
8	.12	−.31***	.29***	.22**	.35***	.53***	.46***	–			
9	.14	−.22**	.44***	−.00	.46***	.49***	.51***	.44***	–		
10	.06	−.45***	.44***	−.03	.50***	.67***	.46***	.49***	.41***	–	
11	.09	−.42***	.47***	.06	.73***	.83***	.72***	.72***	.73***	.78***	–

1—Positive coping, 2—negative coping, 3—presence of meaning, 4—search for meaning, 5—autonomy, 6—environmental mastery, 7—personal growth, 8—positive relations with others, 9—purpose in life, 10—self-acceptance, 11—total PWB score

*** *p* < .001; ** *p* < .01; * *p* < .05

Results showed that positive coping positively correlated with presence and search. Negative coping was negatively correlated with presence, but not with search. As regards relations between religious coping and PWB, there were no significant associations between positive coping and dimensions of PWB and its total score. However, negative correlations were found between negative coping and all the dimensions of PWB: autonomy, environmental mastery, personal growth, positive relations with others, PIL, and self-acceptance.

A different view concerns relations between meaning in life and PWB. Positive associations were found between presence and all the dimensions of PWB and the total score. In contrast, there were only two statistically significant correlations between search and subscales of PWB—search positively correlated with personal growth and positive relations with others. No correlation was found between search and the total PWB score.

**Fig. 1:**
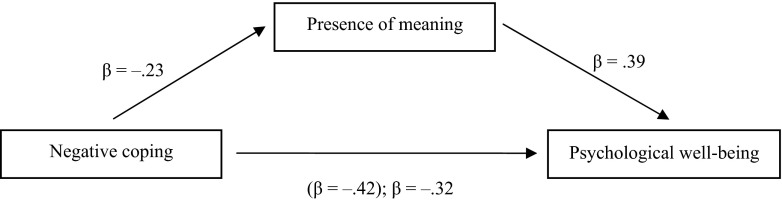
Presence of meaning as mediating the association between negative coping and psychological well-being

In the next step, multiple regression was used to assess mediational effects for religious coping, meaning in life and PWB (Fig. 1). We first established that the conditions for mediation were met (Baron and Kenny 1986). There was no significant association between positive coping and the total score of PWB which invalidates further statistical analyses. However, negative coping was negatively related to PWB which satisfies the first requirement (*β* = −.42; *p* < .001). Fulfilling the second requirement, negative coping was also related to presence of meaning (*β* = −.23; *p* < .01). Satisfying the third requirement, presence was related to PWB, controlling for negative coping (*β* = .39; *p* < .001). The next requirement was that the relation between negative coping and PWB be significantly smaller, with presence entered as a predictor. However, the relation between negative coping and PWB decreased (from −.42 to −.32), although it remained significant. The Sobel test was significant (*z* = −4.30; *p* < .001). Presence of meaning seemed thus to be a partial mediator of the relation between negative coping and PWB.

The mediational effects of presence were also statistically significant for the associations between negative coping and all the dimensions of PWB: autonomy, environmental mastery, personal growth, positive relations with others, PIL, and self-acceptance. The Sobel test results confirmed that presence was a partial mediator between negative coping and the dimensions of psychological well-being.

The results of the further analyses did not allow for testing search for meaning in life as a mediator, because there was no significant association between negative coping and search (*β* = −.06; *p* < .423).

In summary, the results obtained in Study 1 indicate that religious coping is associated with meaning in life. Positive coping positively correlated with both dimensions of meaning in life: presence and search, whereas negative coping was only negatively linked with presence. The results confirmed the first hypothesis showing that religiousness understood in terms of coping styles relates to a sense of purpose and significance. Only negative coping was related negatively to PWB in the scope of the total score and all the dimensions: autonomy, environmental mastery, personal growth, positive relations with others, PIL, and self-acceptance. It allows us to partially verify the second hypothesis indicating associations between religious coping and PWB. The associations only occur in the negative forms of religious coping. The main result of Study 1 was revealing presence of meaning in life as a mediator between negative coping and PWB in the scope of a total score and all its dimensions. However, meaning only partially mediated the above relation which suggests that people’s expectations for significance and purpose in the future cannot be explained completely by religion’s contribution to their meaning in life. This view was supported by the fact that search for meaning in life did not mediate the relation between religious coping and PWB. The third hypothesis was thus partially confirmed.

The results provide support for the mediational role of presence of meaning in life in the relation between religious coping and PWB. Yet, one limitation of this study was that the measure of presence of meaning in life was assessed in only one dimension. Therefore, using a more refined measure of presence appears reasonable and fully justified. In Study 2, we sought to continue to examine the relations between religious coping and PWB by using a multidimensional measure of meaning in life which also has a slightly different character.

## Study 2

The implications of Study 1 are limited by the use of only the one-dimensional presence of meaning, while research suggests that meaning in life can consist of more dimensions (Reker 2005; Wong 1998). Therefore, in Study 2, we sought to expand the assessment of relations between religious coping and PWB by including personal meaning that is based on an implicit theories approach and regarded as “comprising an ideally meaningful life” (Wong 1998). It presents meaning in life in seven domains: achievement, relationship, religion, self-transcendence, self-acceptance, intimacy, and fair treatment. Consistent with the meaning-as-mediator model, we hypothesized that personal meaning would mediate the relation between religious coping and PWB.

### Method

#### Participants

One hundred and eighty-two participants (94 women and 88 men) were given a questionnaire packet consisting of three methods. Ages ranged from 21 to 63, with a mean age of 41 years (SD = 10.21). There were no statistically significant differences in terms of age between women and men. The vast majority of participants were Christians (92.3 %), and only 7.7 % described themselves as non-believers. The participants represented different professions and came from both urban and rural environments. The study was anonymous.

#### Measures

Three psychological tests were administered: Brief RCOPE, the PWB scale and the PMP. All of them were Polish versions. The first two scales were described in Study 1.

The PMP (Wong 1998) measures *implicit meaning* that is viewed as “comprising an ideally meaningful life”. The scale assesses the level of meaning reported by respondents in seven domains: achievement, relationship, religion, self-transcendence, self-acceptance, intimacy, and fair treatment. A total score can also be calculated to reflect overall levels of personal meaning. The Polish version was adapted by Krok (2010).

### Results and Discussion

In the initial examination of the predicted relations, correlations were calculated among religious coping, dimensions of personal meaning, and a total score of PWB, and these are shown in Table 2.

**Table 2:** Correlations among religious coping, personal meaning, and psychological well-being

	1	2	3	4	5	6	7	8	9	10	11
1	–										
2	.18*	–									
3	.22**	−.19**	–								
4	.22**	−.25***	.52***	–							
5	.79***	.01	.39***	.39***	–						
6	.40***	−.18**	.81***	.50***	.59***	–					
7	.26***	−.27***	.53***	.47***	.42***	.50***	–				
8	.21**	−.17*	.41***	.51***	.33***	.33***	.45***	–			
9	.30***	−.20**	.44***	.39***	.43***	.47***	.53***	.44***	–		
10	.48***	−.24**	.78***	.71***	.70***	.81***	.74***	.68***	.74***	–	
11	.08	−.41***	.58***	.31***	.24***	.47***	.35***	.43***	.38***	.54***	–

1—Positive coping, 2—negative coping, 3—achievement, 4—relationship, 5—religion, 6—self-transcendence, 7—self-acceptance, 8—intimacy, 9—fair treatment, 10—total personal meaning score, 11—total psychological well-being score

*** *p* < .001; ** *p* < .01; * *p* < .05

Positive correlations were found between positive coping and personal meaning and its dimensions: achievement, relationship, religion, self-transcendence, self-acceptance, intimacy, and fair treatment. On the contrary, negative coping was negatively associated with personal meaning and all its dimensions except religion. The last result was rather surprising if we take into account opposite directions between positive and negative coping. PWB positively correlated with personal meaning and all its dimensions.

In order to examine more thoroughly relations between personal meaning and PWB, correlations were run between their dimensions. The results are presented in Table 3.

**Table 3:** Correlations between dimensions of personal meaning and psychological well-being

Psychological well-being	Personal meaning							
	Ach	Rel	Reli	ST	SA	Int	FT	IM
Autonomy	.36***	.13	.08	.29***	.21**	.18*	.09	.25***
Environmental mastery	.40***	.19**	.10	.29***	.32***	.35***	.27***	.37***
Personal growth	.56***	.18*	.23**	.42***	.31***	.33***	.44***	.48***
Positive relations with others	.43***	.43***	.23**	.34***	.24***	.46***	.32***	.47***
Purpose in life	.50***	.22**	.30***	.50***	.18*	.24***	.17*	.41***
Self-acceptance	.37***	.28***	.16*	.29***	.34***	.40***	.44***	.44***
Total score	.58***	.31***	.24***	.47***	.35***	.43***	.38***	.54***

Ach, achievement; Rel, relationship; Reli, religion; ST, self-transcendence; SA, self-acceptance; Int, intimacy; FT, fair treatment; IM, total personal meaning score

*** *p* < .001; ** *p* < .01; * *p* < .05

The results showed that there are strong associations between dimensions of personal meaning and PWB as most relationships turned out to be statistically significant. The only associations which were insignificant occurred between relationship and autonomy, religion and both autonomy and environmental mastery, fair treatment and autonomy. All the other relations were significant and positive.

In a similar way as in Study 1, we again used multiple regression to test whether personal meaning (total score) mediated the relation between religious coping and PWB. To assess whether the conditions for mediation were met, we separately assessed each necessary relation (Baron and Kenny 1986). No significant association was found between positive coping and the total score of PWB (*β* = .08; *p* < .26) which does not allow further statistical analyses.

**Fig. 2:**
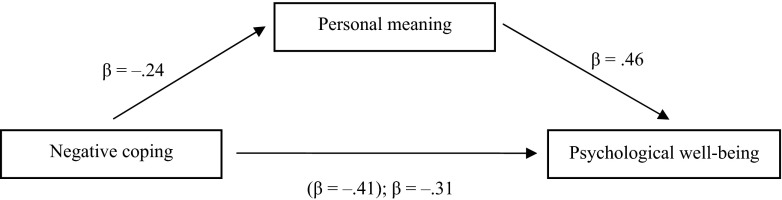
Personal meaning as mediating the association between negative coping and psychological well-being

However, negative coping was negatively related to PWB which meets the first requirement (*β* = −.41; *p* < .001) (Fig. 2). Satisfying the second requirement, negative coping was also related to personal meaning (*β* = −.24; *p* < .001). According to the third requirement, personal meaning was related to PWB, controlling for negative coping (*β* = .46; *p* < .001). Regarding the fourth requirement of mediation, negative coping was still significantly related to PWB (*β* = −.31; *p* < .001) when personal meaning was added to the model. The relation between negative coping and PWB decreased (from −.41 to −.31), although it remained significant. Next, a Sobel test was performed to determine whether personal meaning partially mediated the relation between negative coping and PWB. The Sobel test was significant (*z* = −2.95; *p* < .003), indicating that personal meaning is a partial mediator accounting for at least a portion of the relation between negative coping and PWB.

In order to examine further mediational effects, we conducted statistical analyses for the associations between negative coping and PWB with all the personal meaning dimensions as mediating variables. The results showed that the following dimensions: achievement, relationship, self-transcendence, self-acceptance, intimacy, and fair treatment met the statistical requirements (regressions and a Sobel test) for mediation and were partial mediators in the relations between negative coping and PWB. Religion was the only factor that did not satisfy mediational requirements.

The results obtained in this study fit with the previous ones. Religious coping styles were associated with personal meaning. Positive correlations were found between positive coping and personal meaning and negative correlations between negative coping and personal meaning. All the dimensions of personal meaning showed positive links with PWB. The mediational analyses revealed that personal meaning was a partial mediator between negative coping and PWB which confirms our predictions. In line with results from the previous study, this finding added strong support for the hypothesis that presence of meaning in life is an important partial mediator of the relation between religious coping and PWB, but only for the form of coping which is based on negative appraisals and accusatory attitudes towards God.

## General Discussion

The aim of the studies was to empirically test assumptions that meaning in life may be a mediator in the relation between religious coping and PWB. In addition, we wanted to assess relationships between religious coping, meaning in life, and PWB. These questions were examined by using different indices of meaning in life: presence, search, and personal meaning which enabled us to understand more deeply how individuals form a meaningful life and approach a domain of significance and purpose. The results indicate that these relations exist which has potentially important implications.

Mediational analyses revealed that presence of meaning in life is a partial mediator of the relation between negative religious coping and PWB. Several previous studies implied that meaning in life was a mediator between religion and well-being (Chamberlain and Zika 1992), intrinsic religious orientation and subjective well-being (Ardelt 2003), and religiousness and life satisfaction (Steger and Frazier 2005). The evidence from the present studies indicates that meaning in life plays a mediational role between a negative form of religious coping and PWB. Taken together, these results demonstrate that meaning in life is an important element of religion that imbues religious behaviour with a sense of purpose and significance. It is especially noticeable in the context of religious coping that is strongly connected to the domain of meaning (Pargament et al. 2004; Park 2005, 2013). Approaching challenging and problematic life circumstances, individuals draw on the orienting system of religion and meaning through which they interpret and handle stressful situations. Religious coping methods are used to strengthen other non-religious psychological resources to enable people to face life challenges.

There are two questions that can arise in the context of the current research: why presence of meaning in life turned out to be a partial mediator and why only negative coping was related to PWB. According to recent research, meaning is a central element of religion because every religion addresses important questions related to a sense of purpose and significance (Hood et al. 2009; Park 2013). People turn to religious beliefs and activities in order to find meaning in complex and incomprehensible events. Religious interpretations enable individuals to perceive daily experiences in terms of universal goals and provide explanations for situations of high ambiguity and threat. Therefore, religion is inseparably interconnected with meaning on both the structural and functional levels. As a result, meaning in life only partially mediates the relation between religious coping and PWB mainly because the religious realm is already imbued with meaning.

Analyses of the question why only negative religious coping was related to PWB need to take into account the fact that the relation had a negative character. The less individuals use negative coping strategies, the more their level of PWB increases. This result can suggest that avoidance of negative religious coping strategies such as punishing God appraisals, interpersonal religious discontent, or questioning God’s powers is beneficial for one’s PWB. When facing stressful situations, people rely on a system of religious beliefs, practices, and relationships which affects the ways in which they deal with difficult situations (Pargament 1997). This religious orienting system is translated into concrete situation-specific appraisals which are used to gain or search for a sense of significance. The process enables people to find meaning which, in turn, increases their PWB.

The results of this study also deepen our understanding of the function of religion in both human life as a whole and creating well-being. Previous studies have established that religion is linked to subjective well-being and that meaning in life mediates their relations (Chamberlain and Zika 1992; Krok 2010; Steger and Frazier 2005). The current research showed that religious coping is moderately related to PWB and that meaning in life is a partial mediator in the relation between both factors. These moderate relationships can imply that people do not primarily engage in religious activities to elevate subjective or PWB. Religion contributes to well-being by providing individuals with a sense of meaning, but the main function of religion is to bring people into greater contact with sacred matters, rather than maximizing happiness or self-fulfilment.

The findings also provide evidence that meaning in life is a crucial element of religious coping and PWB. People use religious beliefs, feelings, and practices as a part of their meaning system to cope with life’s difficulties and challenges (Pargament et al. 2005; Park 2005). The links existing between religious coping and meaning in life suggest that religion can influence many aspects of meaning by providing an ultimate motivation to all aspects of a person’s life, establishing goals and value systems and helping to instil a deeper sense of meaning in life. It is unquestionably evident within a framework of the meaning making model proposed by Park (2013), which posits that religious and spiritual factors play an important role in modelling the meaning system of many individuals.

Meaning in life appears instrumental in creating PWB. It mainly refers to the dimensions of presence and personal meaning, but not search which did not have any associations with PWB apart from its two dimensions: personal growth and positive relations with others. The results imply that existential convictions regarding a sense of significance and purpose help individuals to attain higher levels of PWB in the domain of autonomy, environmental mastery, personal growth, positive relations with others, and self-acceptance. They also appear to reflect a noticeably human characteristic of striving for meaning in life as a way of increasing the form of well-being that is based on values and engaged with existential challenges of life. People struggle to explore important areas of their life through personal goal systems and PIL.

The present study certainly has limitations which warrant discussion. First, religiousness was only assessed in terms of religious coping which is the degree to which religion is helpful in understanding and dealing with critical life events. Replication of the current results with other measures of religiousness would reveal the associations of other domains of religion (e.g. specific religious beliefs, emotions, or activities) with PWB and broaden our understanding of which factors are most important in those relations. Second, the current work relied wholly on self-report measures of meaning in life. Although it has been the most popular method of measuring this factor, it does not tap the full range of individual differences regarding a sense of purpose and significance (Reker 2005; Steger 2009). Future research could employ experimental procedures of assessing meaning in life. Procedures consisting in inducing thoughts or feelings related to the domain of meaning would enable researchers to formulate more precise causal relations and understand how meaning can change under the influence of various factors. Thirdly, the assessment was done once at a given time which limits chances of investigating potential changes in meaning in life. Introducing longitudinal methods of evaluations e.g. asking participants to keep a diary of meaningful events for a longer time (i.e. a month or even more) would more precisely divulge the process by which meaning is created from life experiences. A final limitation of the present study is that the samples were mainly composed of Christians. It was a consequence of the specific character of the examined population (Poland) in which a vast majority of the people are Christians. Hence, the findings may not generalize to other populations which include other religions.

Despite its limitations, the current study provides evident support for the hypotheses that meaning in life is a partial mediator through which religious coping is associated with PWB and that presence of meaning in life is strongly linked with well-being based on a eudaimonic approach. The programme of two studies reported here lays a strong foundation for further research exploring the role of meaning in life and other related factors in fostering well-being, as well as applying the literature on religion, meaning, and PWB to important human problems.
